# Artificial Intelligence in Pediatric Orthopedics: A Comprehensive Review

**DOI:** 10.3390/medicina61060954

**Published:** 2025-05-22

**Authors:** Andrea Vescio, Gianluca Testa, Marco Sapienza, Filippo Familiari, Michele Mercurio, Giorgio Gasparini, Sergio de Salvatore, Fabrizio Donati, Federico Canavese, Vito Pavone

**Affiliations:** 1 Department of Life Science, Health, and Health Professions, Link Campus University, 00165 Rome, Italy; a.vescio@unilink.it; 2 Department of Orthopaedic and Trauma Surgery, “Mater Domini” University Hospital, “Magna Græcia” University, 88100 Catanzaro, Italy; michele.mercurio@unicz.it (M.M.); gasparini@unicz.it (G.G.); 3 Department of General Surgery and Medical Surgical Specialties, Section of Orthopaedics and Traumatology, Policlinico Rodolico-San Marco, University of Catania, 95123 Catania, Italy; gianluca.testa@unict.it (G.T.); vpavone@unict.it (V.P.); 4 Department of General Surgery and Medical Surgical Specialties, Orthopedics Unit, Bambino Gesù Pediatric Hospital, 00165 Rome, Italy; sergio.desalvatore@gmail.com (S.d.S.); fabrizio.donati@opbg.net (F.D.); 5 Orthopedic and Traumatology Department, IRCCS Istituto Giannina Gaslini, 16147 Genoa, Italy; canavese_federico@yahoo.fr; 6 DISC-Dipartimento Di Scienze Chirurgiche e Diagnostiche Integrate, University of Genova, 16126 Genova, Italy

**Keywords:** artificial intelligence, pediatric orthopedics, machine learning, deep learning, spinal deformities, bone age assessment, fracture detection, developmental dysplasia of the hip, medical imaging, clinical decision support

## Abstract

*Background and Objectives*: Artificial intelligence (AI) has seen rapid integration into various areas of medicine, particularly with the advancement of machine learning (ML) and deep learning (DL) techniques. In pediatric orthopedics, the adoption of AI technologies is emerging but still not comprehensively reviewed. The purpose of this study is to review the latest evidence on the applications of artificial intelligence in the field of pediatric orthopedics. *Materials and Methods*: A literature search was conducted using PubMed and Web of Science databases to identify peer-reviewed studies published up to March 2024. Studies involving AI applications in pediatric orthopedic conditions—including spinal deformities, hip disorders, trauma, bone age assessment, and limb discrepancies—were selected. Eligible articles were screened and categorized based on application domains, AI models used, datasets, and reported outcomes. *Results*: AI has been successfully applied across several pediatric orthopedic subspecialties. In spinal deformities, models such as support vector machines and convolutional neural networks achieved over 90% accuracy in classification and curve prediction. For developmental dysplasia of the hip, deep learning algorithms demonstrated high diagnostic performance in radiographic interpretation. In trauma care, object detection models like YOLO and ResNet-based classifiers showed excellent sensitivity and specificity in pediatric fracture detection. Bone age estimation using DL models often matched or outperformed traditional methods. However, most studies lacked external validation, and many relied on small or single-institution datasets. Concerns were also raised about image quality, data heterogeneity, and clinical integration. *Conclusions*: AI holds significant potential to enhance diagnostic accuracy and decision making in pediatric orthopedics. Nevertheless, current research is limited by methodological inconsistencies and a lack of standardized validation protocols. Future efforts should focus on multicenter data collection, prospective validation, and interdisciplinary collaboration to ensure safe and effective clinical integration.

## 1. Introduction

The foundations for the application of artificial intelligence (AI) in the medical field were laid as early as 1956. However, significant advancements did not emerge until the 21st century [1]. Among the most transformative developments has been the rise of machine learning (ML), particularly deep learning (DL), which has enabled more sophisticated and powerful medical applications [1]. Deep learning is an advanced form of unsupervised ML that utilizes multiple layers of neural networks to replicate human cognitive functions related to data analysis and decision making. It is commonly referred to as artificial neural networks (ANNs) and can simulate brain-like processing through algorithmic architectures. A deep neural network (DNN) typically includes more than three layers, encompassing both input and output layers [2,3].

In recent years, artificial intelligence large language models (AI LLMs) have rapidly expanded across various domains, including medicine. Notable examples include ChatGPT 3.5 (OpenAI, San Francisco, CA, USA), Gemini 2.0 (Google, Mountain View, CA, USA), and Microsoft CoPilot (Microsoft, Redmond, WA, USA) [3]. Their accessibility, affordability, and broad availability have contributed to a growing number of patient-initiated requests for orthopedic evaluations and consultations via these platforms.

Within healthcare, AI has demonstrated increasing utility in tasks such as medical imaging analysis—encompassing modalities like X-rays, MRI, and CT scans [4,5], personalized treatment plans [6], drug discovery [7], and predictive analytics. The ongoing integration of AI technologies into clinical practice is reshaping diagnostic and therapeutic paradigms, offering improvements in both efficiency and accuracy. Furthermore, recent studies have shown that some AI-based LLMs can provide clinically accurate responses when evaluated by fellowship-trained surgeons and attending physicians.

Orthopedic surgery, as a technologically progressive specialty, has begun integrating AI applications across various subspecialties [8]. In the field of foot and ankle surgery, for instance, several AI models have been developed. However, many of these models lack external validation, limiting their generalizability [9]. Recently, General Orthopedic Artificial Intelligence (GOAI) was proposed as a medical AI system aimed to assist doctors in formulating personalized treatment plans by analyzing patients’ symptoms, medical history and imaging data [10].

Despite growing enthusiasm and promising results, several barriers impede the widespread adoption of AI in orthopedic practice. These include the high costs and time investments required for implementation, inconsistencies in the reliability of AI systems, and a lack of long-term outcome data to assess efficacy and safety [11]. Ethical considerations—particularly regarding patient confidentiality, informed consent, and the handling of large, sensitive datasets—also remain critical challenges that must be addressed [11]. Artificial intelligence (AI) is gaining increasing attention in pediatric orthopedics, with growing awareness among specialists about its clinical and diagnostic potential [11].

Recent studies have demonstrated AI’s ability to classify pediatric spinal radiographs with high accuracy, facilitating the creation of large-scale imaging registries that may enhance research and care delivery [12]. In the context of adolescent idiopathic scoliosis, deep learning models have been used not only for diagnosis but also to support surgical decision making and predict postoperative outcomes [13].

Additionally, AI tools have shown promise in reducing interobserver variability in assessing hip dysplasia indices on ultrasound, highlighting their role in improving diagnostic consistency across expertise levels [14].

Despite its promise, the integration of AI into clinical workflows remains at an early stage, with ongoing concerns about trust, usability, and ethical implications [11].

The purpose of this study is to review the latest evidence on the applications of artificial intelligence in the field of pediatric orthopedics.

## 2. Materials and Methods

A review of the literature was undertaken using the PubMed and Web of Science database with the following research string: “(“Artificial Intelligence”[Mesh] OR “Machine Learning”[Mesh] OR “Deep Learning”[Mesh] OR “Artificial Intelligence” OR “Machine Learning” OR “Deep Learning”) AND (“Pediatrics”[Mesh] OR “Pediatric” OR “Children” OR “Adolescent”) AND (“Orthopedics”[Mesh] OR “Orthopedic Procedures”[Mesh] OR “Bone Diseases”[Mesh] OR “Musculoskeletal Abnormalities”[Mesh] OR “Orthopedic” OR “Musculoskeletal”).

Studies were considered eligible for inclusion if they addressed topics related to artificial intelligence (AI) within the field of pediatric orthopedics. Titles and abstracts were initially screened based on the following inclusion criteria: any level of evidence, English language, publication in peer-reviewed journals, and a focus on clinical outcomes involving AI in pediatric orthopedic care. Excluded were non-English publications, studies centered on other orthopedic subspecialties, duplicates, preclinical or preliminary studies, articles unrelated to the topic, those lacking sound scientific methodology, or without an accessible abstract. Additionally, reference lists of the included studies were manually reviewed to identify other potentially relevant publications.

## 3. Results

**Figure 1:**
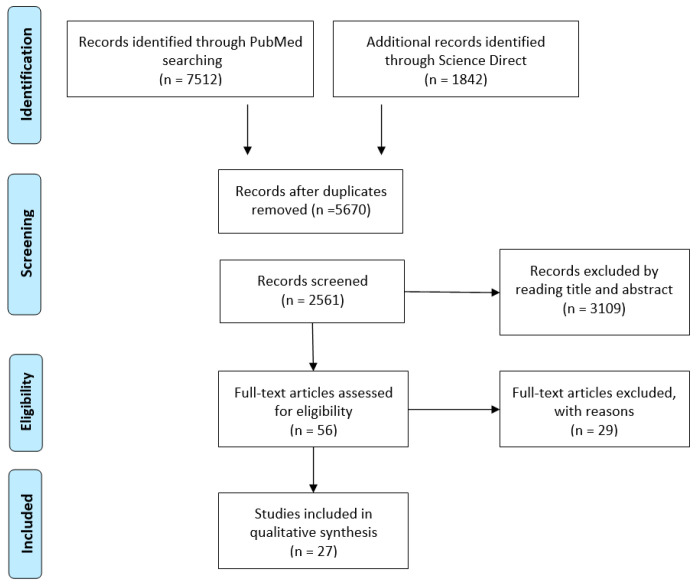
Preferred Reporting Items for Systematic Reviews and Meta-Analysis flowchart of the literature review.

An initial search yielded 9354 articles. After removing duplicates, 2561 articles remained for screening. Based on the previously defined selection criteria, 154 articles were identified as suitable for full-text review. Following this review and a manual examination of reference lists, a total of 27 articles met the final inclusion criteria (Figure 1).

The main pediatric orthopedics themes reported in the literature are Spine deformities, Pediatric trauma, Bone Age assessment, Leg Length Discrepancy, Growing pain, Venous Thromboembolism (Table 1).

### 3.1. Spine Deformities

Mulford et al. [12] reported precision ranging from 0.98 to 1.00 in the AP images, and from 0.91 to 1.00 on the lateral images in AIS classification. Chen et al. [13] demonstrated mean square error of 2.77 × 10^−5^ and an average absolute error of 0.00350 on the validation set in predicting surgical outcomes using deep learning for decision making and outcome prediction. Likewise, Lv et al. [20] applied five different machine learning models to analyze the clinical evolution of AIS and reported an AUC varying between 0.767 (95% confidence interval [CI]: 0.710–0.824) and 0.899 (95% CI: 0.842–0.956).

Kabir et al. [27] validated the use of deep learning for measuring growing rod length in 387 children with early-onset scoliosis who underwent surgical treatment, achieving an average precision (AP) varying between 67.6% and 94.8%. The MAD ± SD of the rod length change was 0.98 ± 0.88 mm, and the ICC _[1,2]_ was 0.90 between the manual and artificial intelligence (AI) adjustment measurements. Additionally, Fraiwan et al. [17] retrospectively assessed scoliosis and spondylolisthesis in 338 pediatric patients using deep transfer learning, reporting accuracy for three-class classification varying between 96.73% and 98.02%. Zhang et al. [33] experimented with the ScolioNets deep learning model, and highlighted the following parameters: Sensitivity 84.88% (75.54–91.70), Negative Predictive Value 89.22% (84.25–93.70), Specificity 67.44% (59.89–74.38), Positive Predictive Value 56.59 (50.81–62.20), Accuracy 73.26% (67.41–78.56). Negrini et al. [36] investigated a clinical parameter, the angle of trunk rotation (ATR), and reported varying accuracies according to the Cobb angle (74, 81, 79, 79, and 84% for 15-, 20-, 25-, 30- and 40-degree thresholds, respectively).

### 3.2. Pediatric Hip Disorders

Wu, Q. et al. [28] retrospectively analyzed 2000 hips utilizing deep-learning and reported the 95% limits of agreement (95% LOA) of the system as −0.93° to 2.86° (bias = −0.03°, *p* = 0.647). Zhang, S.C. et al. [34] reported similar results for acetabular index in non-dislocated and dislocated hips (95% LOA were −3.27–2.94° and −7.36–5.36°, respectively (*p* < 0.001)); in addition, the deep learning model achieved sensitivity of 95.5% and specificity of 99.5% for hip dislocation diagnosis. Xu W. et al. [37] reported their deep learning (Mask-RCNN) experience on retrospectively assessed 1398 x-rays. The authors highlighted Tönnis and International Hip Dysplasia Institute (IHDI) classification accuracies for both hips ranging from 0.86 to 0.95. Ghasseminia et al. [14] implemented an ultrasound-FDA-cleared AI software package (Medo Hip) and calculated interobserver reliability for alpha angle measurements for 12 readers, AI versus subspecialists (ICC = 0.87 for sweeps, 0.90 for single images). AI reliability deteriorated more than human readers for the poorest-quality images.

### 3.3. Pediatric Trauma

Zech et al. [16] introduced a deep learning model capable of identifying fractures in pediatric upper extremity radiographs, with an AUC varying between 0.876 ([0.845–0.908, *p* < 0.001) and 0.844 ([0.805–0.883] with AI, *p* < 0.001). Similarly, Parpaleix et al. [19] developed a combined musculoskeletal and chest deep learning detection system, achieving 90.1% accuracy in emergency settings. In diagnosing supracondylar humerus fractures, the area under the curve (AUC) of anteroposterior and lateral elbow radiographs was 0.65 and 0.72 in the retrospective radiomics-based machine learning study by Yao et al. [32]. Kavak et al. [18] introduced You Only Look Once (YOLO)v8, a convolutional neural network (CNN) model, which assessed 5150 (850 fractures, not fractures 4300) radiographs and reported an accuracy varying between 93 and 95% in detecting fractures. In a previous version of the software, Binh et al. [31] designed a multi-class deep learning model for detecting pediatric distal forearm fractures, based on the AO/OTA classification, which also reached 92% accuracy.

### 3.4. Bone Age Assessment

Tajmir et al. [21] achieved accuracy of 68.2% overall and 98.6% within 1 year variability in bone age interpretation. Rassmann S et al. [24] validated Deeplasia in seven different genetic bone diseases and estimated a test–retest precision similar to a human expert.

### 3.5. Leg Length Discrepancy

Zheng et al. [25] developed a deep learning model to assess leg length discrepancies, achieving a dice similarity coefficient of 0.94. Calculation time for the DL method per radiograph was faster than the mean time for radiologist manual calculation (1 s vs. 96 s ± 7, respectively; *p* < 0.001). Similarly, Kim et al. [26] evaluated a deep learning model that measured bilateral iliac crest height differences, reporting interclass correlations (ICCs) ranging from 0.914 to 0.997 between the deep learning model and radiologists. van der Lelij, T. J. N. et al. [15] investigated a machine learning model assessing the measurement of the femur length, tibia length, full leg length (FLL), leg length discrepancy (LLD), hip-knee-ankle angle (HKA), mechanical lateral distal femoral angle (mLDFA), and mechanical medial proximal tibial angle (mMPTA). The authors valuated 58 legs, aged 11 to 18 years old, 76% of the cases for LLD measurements, 88% for FLL and femur length, 91% for mLDFA, 97% for HKA, 98% for mMPTA, and 100% for tibia length. Zech JR et al. [35], in a region-based Convolutional Neural Network Retrospective study trial, highlighted absolute errors of AI measurements of the femur, tibia, and lower extremity in the test data set of 0.25, 0.27, and 0.33 cm, respectively.

### 3.6. Other Pediatric Orthopedic Conditions

In a study on growing pains, Akal et al. [22] applied machine learning techniques to diagnose and achieved 0.99 sensitivity, 0.97 specificity, 0.98 accuracy in differentiating growing pains from other musculoskeletal conditions.

Papillon et al. [23] developed a machine learning algorithm to predict VTE in injured children, with a baseline rate of VTE (0.15%) having a predicted rate of 0.01–0.02% and 1.13–1.32% for low and high risk, respectively.

Hou, T. et al. [29] investigated non-accidental trauma in pediatric trauma patients using supervised machine learning and reported a specificity of 99.94 and sensitivity of 36.59 for confirmed NAT, and specificity of 99.93 and sensitivity of 70.12 for suspected NAT.

## 4. Discussion

Numerous studies in the literature have highlighted the advantages of AI in pediatric orthopedics. The primary areas where AI has been applied include spinal and hip disorders, pediatric trauma, bone age assessment, and leg length discrepancy.

Several researchers have explored the use of AI in assessing the severity and predicting the progression of adolescent idiopathic scoliosis (AIS). Notably, the measurement of the Cobb angle varies between operators by approximately 4° to 8° [38], underscoring the potential of AI as a valuable tool in AIS classification. Mulford et al. [12] described a great precision (0.98 to 1.00 in the AP images, and from 0.91 to 1.00 on the lateral images) in AIS classification. Fraiwan et al. [17] reported an accuracy classification varying between 96.73% and 98.02% for scoliosis and spondylolisthesis, respectively. On the ScolioNets database, proposed by Zhang et al. [34], the diagnosis accuracy was 73.26% (67.41–78.56).

However, despite its promise, identifying the most effective AI-based tool remains challenging. The variability in assessment methods, along with discrepancies in accuracy, specificity, and sensitivity values, prevents a definitive conclusion regarding the superiority of AI over human operators in clinical practice.

Skeletal maturity quantification is not simple. In fact, it has been defined as a complex and multifactorial disease, and a single index could be insufficient to predict its evolution [39]. Numerous progression-associated indexes were identified for the AIS, and researchers proposed a therapeutic strategy based on reliable and reproducible algorithms [39,40]. Lv et al. [20] introduced five models of machine learning with an AUC varying between 0767 and 0.899; others reported a range between 67.6% and 94.8% in the prognosis of early-onset scoliosis. It would be especially desirable to integrate AIS progression prediction and bone age assessment software. Although the analyzed findings were not found to be superior to a human operator (accuracy was 68.2% overall), the results of the Deeplasia tool could be of particular concern. In fact, the AI software precision was similar to that of a human expert in the assessment of seven different genetic bone diseases, and for this reason, it is reasonable to assume that it could be a powerful weapon in the hand of general and less experienced radiologists [24].

Regarding the DDH, there are no universally accepted guidelines for development screening [41]. In Europe, ultrasound screening is categorized into two approaches: selective, implemented in German-speaking countries, Italy, Slovenia, and Slovakia, and universal, adopted in the remaining regions [42]. In several countries, pelvic radiographs serve as the primary diagnostic modality. The severity of DDH is commonly assessed using the Tönnis Classification (Grades 1–4), which evaluates the relative positioning of the ossific nucleus and acetabulum [14]. Additionally, the acetabular index, measured via Hilgenreiner’s line through the triradiate cartilages, constitutes a fundamental assessment parameter [14].

The reliability and the standardization of the image is the limit of the methods. In a review, after the analysis of more than 130 articles, the authors reported that only 51.9% presented correct sonographic images according to Graf’s criteria [43]. Similar limits are related to the execution of pelvis X-rays in children. The evaluated studies reported good results in ultrasound and radiological instrumentation, but the ability of AI in the interpretation of low-quality images is not clear. Relevant data were reported by Parpaleix et al. [19]. In fact, AI demonstrated similar results for the different body regions, except for the ribs. In this case, AI had difficulty detecting minor or subtle fractures. The YOLO tool was investigated in two articles, reporting accuracy superior to 90% of cases.

The diagnosis of growing pains is based on the criteria described by Peterson, which include (1) intermittent pain occurring once or twice weekly, typically in the late afternoon or at night, with pain-free intervals during the day; (2) non-articular pain predominantly affecting the shins, calves, thighs, or popliteal region, usually bilateral; and (3) resolution of symptoms by the following morning without any objective signs of inflammation [44].

Although this clinical presentation may appear straightforward, it is essential to rule out other conditions that can mimic similar symptoms, such as trauma, neoplasms, or infections, as these may lead to delayed or incorrect diagnoses [45]. In this context, Akal et al. [22] demonstrated promising results in the use of machine learning techniques for the diagnosis of growing pains, suggesting that AI may serve as a valuable tool in pediatrics and general orthopedic diagnostics. Conversely, diagnosing non-accidental trauma (NAT) remains significantly more complex. The World Health Organization identifies child abuse and neglect as a serious global public health concern [46]. While several validated screening tools exist, no universally accepted questionnaire is currently available for the early detection of child abuse. Although combining multiple assessment tools may improve diagnostic accuracy, such approaches are often time-consuming and not consistently feasible in routine clinical settings [46]. Of particular note, Hou et al. [29] reported AI-driven diagnostic performance for NAT with a specificity approaching 100% and a sensitivity of 70.12%, highlighting its potential utility in this challenging field.

Currently, there is no consensus regarding the number of radiographic images or other imaging modalities required for AI training, validation, and assessment. The number of patients included in AI studies varies widely, from 250 to 10,813. Given these inconsistencies, collaboration between governmental institutions and supranational pediatric orthopedic societies is essential to establish guidelines for the standardization and acceptability of AI tools.

Furthermore, multiple AI technologies have been proposed for pediatric orthopedics, but the most appropriate approach remains unclear. Deep learning and convolutional neural networks (CNNs) enable machines to engage in cognitive processes but are inherently limited by the intelligence of their developers [47]. AI’s ability to interpret and make decisions based on real-world perception, reasoning, learning, and environmental interactions remains uncertain [47].

A recent survey reported that only one-third of orthopedic respondents believe AI is not dangerous, while the majority expressed concerns about privacy and safety [48].

On the other hand, concerns have been raised regarding the future of pediatric orthopedic education and training. AI technologies should complement rather than replace human expertise, as excessive reliance on AI may diminish critical clinical reasoning and decision-making skills among practitioners. Recent findings highlighted that the incorporation of AI LLMs in pediatric orthopedics could offers substantial potential advantages and can significantly boost and advance the level of patient care; on the other hand, ethical issues should be effectively addressed [49]. Moreover, there is a generally low familiarity with AI among pediatric orthopedic surgeons, and the data suggest that comprehensive educational programs be developed and offered that explore AI in healthcare and address common concerns [11]. The use of AI LLMs in this field remains limited due to the absence of comprehensive databases and insufficient training on specialized medical data [50]. As a result, models such as ChatGPT may generate incorrect responses, making it challenging for students to identify and correct such errors [50].

One of the most critical ethical concerns is equitable access to AI. AI-driven healthcare solutions should not be exclusively available to affluent regions or institutions. In order to guarantee that underprivileged communities also benefit from AI, it is imperative that policymakers and healthcare organizations collaborate [49].

Finally, concerns have been raised regarding the unethical use of AI-generated content, highlighting the need for transparency, integrity, and guidelines to prevent misinformation and manipulation of decision making [51]. The potential impact of AI-generated content on research practices and publication ethics further underscores the necessity of well-defined policies and regulatory oversight.

## 5. Conclusions

Numerous artificial intelligence tools have been developed in the field of pediatric orthopedics, with key applications in spinal and hip disorders, pediatric trauma, bone age assessment, and leg length discrepancy. While AI has proven to be a valuable tool in pediatric orthopedics, it has not demonstrated superiority over human operators in direct comparisons. Ethical concerns, transparency issues, and policy debates remain significant challenges. Collaboration between governmental institutions and supranational pediatric orthopedic societies is essential for the development of standardized guidelines and protocols.

## Figures and Tables

**Table 1:** Summary of study results. AIS, adolescent idiopathic scoliosis; EOS, early-onset scoliosis; SPL, spondylolisthesis; HIP, hip disorders; LLD, leg length discrepancy; BAA, bone age assessment; GP, growing pain; VTE, venous thromboembolism; CNN, artificial neural network classification; ML, machine learning; DL, deep learning.

#	Authors (Year)	Medical Condition	AI	Population	Results
1	van der Lelij, T. J. N. et al. (2024) [15]	LLD	ML	58 legs	76% of the cases for LLD measurements, 88% for FLL and femur length, 91% for mLDFA, 97% for HKA, 98% for mMPTA, and 100% for tibia length.
2	Zech JR et al. (2024) [16]	Trauma	DL	240 upper extremity fractures	AUC varying between 0.876 ([0.845–0.908, *p* < 0.001) and 0.844 ([0.805–0.883] with AI, *p* < 0.001)
3	Fraiwan M et al. (2022) [17]	AIS and SPL	DL	338 patients	accuracy for three-class classification varying between 96.73% and 98.02%
4	Kavak N. et al. (2024) [18]	Trauma	CNN	5150 radiographs	accuracy varying between 93 and 95% in detecting fractures
5	Parpaleix A et al. (2023) [19]	Trauma	DL	1772 patients, musculoskeletal and chest detection	Accuracy was 90.1%
6	Lv Z et al. (2023) [20]	AIS	ML	1581 patients	AUC: 0.767–0.899
7	Tajmir SH et al. (2019) [21]	BAA	ML	280 patients	Accuracy was 68.2% overall and 98.6% within 1 year.
8	Akal F et al. (2022) [22]	GP	ML	398 patients	0.99 sensitivity, 0.97 specificity, 0.98 accuracy,
9	Papillon SC et al. (2023) [23]	VTE	ML	383,814 Patients	Baseline rate of VTE (0.15%) with a predicted rate of 0.01–0.02% and 1.13–1.32% for low and high risk, respectively
10	Rassmann S et al. (2024) [24]	BAA	CNN	568 radiographs from 189 patients with molecularly confirmed diagnoses of seven different genetic bone disorders	98.5% accuracy on the test set of the Radiological Society of North America
11	Zheng Q et al. (2020) [25]	LLD	DL	179 patients	(Dice similarity coefficient, 0.94). Mean absolute error ([MAE], 0.45 cm), full pediatric leg lengths (r = 0.99; MAE, 0.45 cm), and full LLD (r = 0.92; MAE, 0.51 cm)
12	Kim MJ et al. (2022) [26]	LLD	DL	300 patients	Interclass correlations (ICCs) ranged from 0.914 to 0.997. The mean absolute error was 2.3 ± 5.2 mm.
13	Mulford et al. (2024) [12]	AIS	DL	7777 AP images and 5621 lateral images	Precision ranged from 0.98 to 1.00 in the AP images, and from 0.91 to 1.00 on the lateral images for classification of pediatric spinal disorders.
14	Kabir, M. H. et al. (2025) [27]	EOS	DL	387 patients	Average precision (AP) varying between 67.6% and 94.8%. The MAD ± SD of the rod length change was 0.98 ± 0.88 mm, and the ICC was 0.90 between the manual and artificial intelligence (AI) adjustment measurements.
15	Chen, K. et al. (2025) [13]	AIS	DL	425 patients	Mean square error of 2.77 × 10^−5^ and an average absolute error of 0.00350 on the validation set
17	Wu, Q. et al. (2023) [28]	HIP	DL	1000 patients	The 95% limits of agreement (95% LOA) of the system were −0.93° to 2.86° (bias = −0.03°, *p* = 0.647).
18	Hou, T. et al. (2025) [29]	NAT	ML	364,217 patients	Confirmed NAT specificity 99.94, sensitivity 36.59, Suspected NAT specificity 99.93, sensitivity 70.12
19	Shelmerdine, S. C. et al. (2024) [30]	Trauma	ML	500 patients	Protocol aims for 92% accuracy in pediatric fracture detection.
20	Binh, L. N. et al. (2024) [31]	Trauma	DL	An 88-image distal forearm fracture dataset	92% accuracy in distal forearm fracture detection.
21	Yao, W. et al. (2024) [32]	Trauma	ML	411 supracondylar humerus fractures	The area under the curve (AUC) of anteroposterior and lateral elbow radiographs is 0.65 and 0.72.
22	Zhang et al. (2023) [33]	AIS	DL	2158 patients	Sensitivity 84.88% (75.54–91.70), negative predictive value 89.22% (84.25–93.70), specificity 67.44% (59.89–74.38), positive predictive value 56.59 (50.81–62.20), accuracy 73.26% (67.41–78.56)
23	Zhang, S. C. et al. (2020) [34]	HIP	DL	1138 patients grouped according to age and into ‘dislocation’ and ‘non-dislocation’	Sensitivity 95.5% and specificity 99.5% for dislocation. Bland–Altman 95% limits of agreement for acetabular index, non-dislocated and dislocated hips were −3.27–2.94° and −7.36–5.36°, respectively (*p* < 0.001).
24	Ghasseminia et al. (2022) [14]	HIP	ML	240 hips	AI versus subspecialists (ICC = 0.87 for sweeps, 0.90 for single images)
25	Zech JR et al. (2024) [35]	LLD	CNN	523 patients	Absolute errors of AI measurements of the femur, tibia, and lower extremity in the test data set were 0.25, 0.27, and 0.33 cm, respectively
26	Negrini et al. (2023) [36]	AIS	ML	10,813 patients	Accuracies of 74, 81, 79, 79, and 84% for 15-, 20-, 25-, 30- and 40-degree thresholds for predicting AIS evolution
27	Xu W. et al. (2022) [37]	HIP	CNN	1398 x-rays	Tönnis and International Hip Dysplasia Institute (IHDI) classification accuracies for both hips ranged from 0.86 to 0.95
